# Taste and smell perception and quality of life during and after systemic therapy for breast cancer

**DOI:** 10.1007/s10549-018-4720-3

**Published:** 2018-02-23

**Authors:** Y. C. de Vries, S. Boesveldt, C. S. Kelfkens, E. E. Posthuma, M. M. G. A. van den Berg, J. Th. C. M. de Kruif, A. Haringhuizen, D. W. Sommeijer, N. Buist, S. Grosfeld, C. de Graaf, H. W. M. van Laarhoven, E. Kampman, R. M. Winkels

**Affiliations:** 1 Division of Human Nutrition, Wageningen University, Wageningen, The Netherlands; 2 Top Institute Food and Nutrition, Wageningen, The Netherlands; 3 Department of Health Sciences, VU University Amsterdam, Amsterdam, The Netherlands; 4 Ziekenhuis Gelderse Vallei, Ede, The Netherlands; 5 Department of Oncology, Academic Medical Center, Amsterdam, The Netherlands; 6 Flevoziekenhuis, Almere, The Netherlands; 7 Amphia Ziekenhuis, Breda, The Netherlands; 8 Department of Oncology, Alexander Monro Ziekenhuis, Bilthoven, The Netherlands; 9 Department of Public Health Sciences, Penn State College of Medicine, Hershey, PA USA

**Keywords:** Breast cancer, Quality of life, Chemotherapy, Herceptin, Trastuzumab, Taste, Taste loss, Dysgeusia, Smell

## Abstract

**Purpose:**

The purpose of the study was to assess self-reported taste and smell perception after chemotherapy in breast cancer patients compared with women without cancer, and to assess whether taste and smell perception is associated with quality of life after the end of chemotherapy.

**Methods:**

We included 135 newly diagnosed breast cancer patients who completed chemotherapy and 114 women without cancer. Questionnaires on taste, smell, and quality of life were completed shortly after and 6 months after chemotherapy (patients) or at two moments with 6 months’ time window in between (comparisons).

**Results:**

Self-reported taste and smell perception were significantly lower in patients shortly after chemotherapy compared to the comparison group. Most patients recovered 6 months after chemotherapy, although patients who were still receiving trastuzumab then reported a lower taste and smell perception compared to patients who were not. A lower self-reported taste and smell were statistically significantly associated with a worse quality of life, social, emotional, and role functioning shortly after chemotherapy. Six months after chemotherapy, taste and smell were statistically significantly associated with quality of life, social and role functioning, but only in patients receiving trastuzumab.

**Conclusions:**

Most taste and smell alterations recovered within 6 months after the end of chemotherapy for breast cancer, but not for patients receiving trastuzumab. These results highlight the importance of monitoring taste and smell alterations during and after treatment with chemotherapy and trastuzumab, as they may impact quality of life.

## Introduction

Taste and smell alterations are amongst the most distressing side effects of chemotherapy treatment in cancer patients and may seriously impact everyday life of cancer patients [1]. Qualitative studies show that taste and smell alterations during chemotherapy have an impact on patient’s lives in terms of household roles (e.g., partners that take over grocery shopping and cooking) and social interactions (e.g., not eating out or inviting friends for dinner) [2, 3]. Also quantitative studies have shown that cancer patients with an altered taste and/or smell during chemotherapy have a lower quality of life [4–7].

Previous research has shown that taste and smell alterations are largely transient, and usually recover within the first three months after the end of chemotherapy [8–10]. However, some studies suggest that taste and smell may be distorted well beyond the end of chemotherapy [11–13]. To date, there is not much known about factors that influence taste and smell perception after the end of chemotherapy, but potentially this is affected by the consequent treatment that patients receive. E.g., breast cancer patients treated with neo-adjuvant chemotherapy may still undergo surgery and/or radiotherapy, about 60–75% of patients receive hormonal therapy [14] and approximately 30% of patients receives trastuzumab [15, 16]. If, and to what extent these factors relate to taste and smell perception, and whether it is related to quality of life after the end of chemotherapy is currently unknown.

To understand more about the nature and impact of taste and smell changes after chemotherapy treatment, the aim of the current study was twofold. First, we assessed reported taste and smell changes shortly after, and 6 months after chemotherapy in breast cancer patients compared to a group of women without breast cancer. Second, we aimed to determine the association between taste and smell perception and quality of life shortly after and 6 months after chemotherapy.

## Materials and methods

### Participants

This study is part of the COBRA-study [17], an observational multi-center study among breast cancer patients during chemotherapy and a comparison group of women without cancer of similar age. Women with newly diagnosed, stage I–IIIB, operable breast cancer, who were scheduled to receive chemotherapy were recruited for this study in 11 hospitals in the Netherlands. In addition, we recruited a comparison group of women without breast cancer. Participants for this comparison group were recruited via patients; patients were asked to distribute envelopes with study information to friends, acquaintances, and colleagues of similar age. If these friends/acquaintances/colleagues were interested in participating in the study, they were asked to contact the researchers who explained the study procedures. To be eligible for the patient or comparison group, participants had to be at least 18 years old and had to able to communicate in Dutch. For both groups, exclusion criteria were as follows: history of cancer, previous treatment with chemotherapy, pregnancy or the intention to get pregnant during the study period, dementia or other mental conditions that made it impossible to comply with study procedures. The protocol was approved by the Medical Ethical Committee of Wageningen University, Wageningen, the Netherlands. All participants provided written informed consent before enrolment.

### Study design

We assessed self-reported taste and smell perception and quality of life at two moments. For breast cancer patients, this was within 1 month after the last chemotherapy cycle (T1) and approximately 6 months after the last chemotherapy cycle (T2). Most of the patients who received trastuzumab as part of their systemic treatment were still receiving this on T2 (*n* = 29 out of *n* = 31 patients receiving trastuzumab), but had completed the chemotherapy part of systemic treatment at T1.

**Fig. 1:**
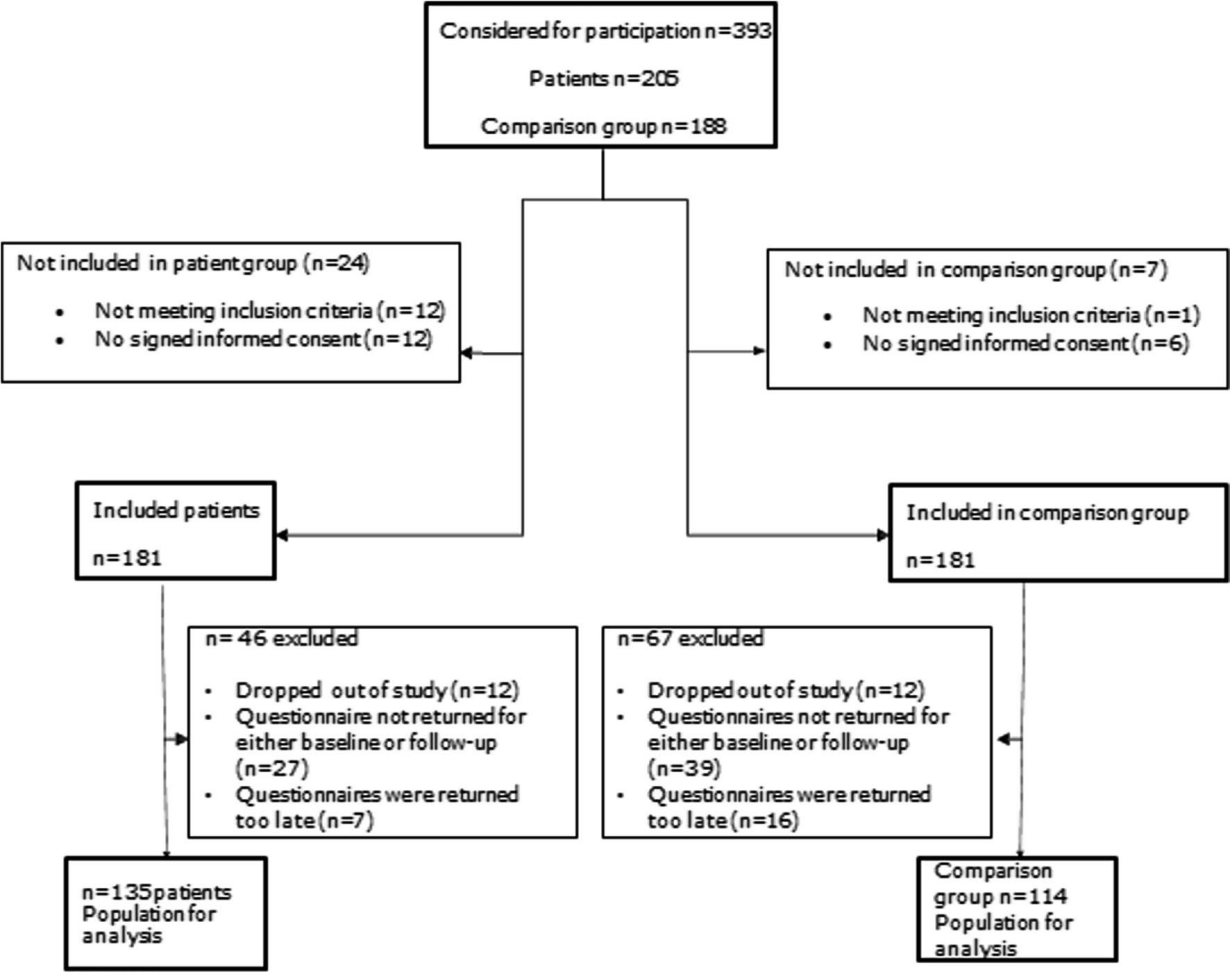
Flow chart of participants of the COBRA study, an observational multi-center study among breast cancer patients during chemotherapy and a comparison group of women without cancer of similar age

In the comparison group, measurements took place at two moments that were approximately 6 months apart. In total, 135 patients and 114 controls were included in the analyses, see Fig. 1 for an overview flow chart of in- and excluded participants. Patients who did not fill in the questionnaires within 1 month after the last chemotherapy cycle (T1), or did not fill in the questionnaires within 5–8 months after the last chemotherapy cycle (T2) were excluded from the analyses (*n* = 7). On average, the time between the first and second measurement was 207 ± 18 days (patients) and 194 ± 17 days (controls).

## Measurements

### Self-reported taste and smell

The appetite, hunger, and sensory perception (AHSP) questionnaire was used to assess self-judgement of taste and smell perception [18]. The questionnaire consists of questions answered on a 5 point Likert scale. For this study, we used the taste (8 items, score range 8–40) and smell (6 items, range 6–30) scale. A higher score corresponds to a more positive judgement about current taste and smell perception.

To assess the prevalence of taste and smell changes in patients, two questions were added from the 16-item taste and smell questionnaire [19]: (1) Have you noticed any changes in your sense taste compared to before chemotherapy? (2) Have you noticed any changes in your sense smell compared to before chemotherapy? Answer possibilities: no, it is the same; yes, it is better; yes, it is worse.

### Quality of life

The EORTC QLQ-C30 was used to assess health-related quality of life [20]. For this study, we used the scales for global quality of life, and the functional scales for social, role and emotional functioning. Questions were asked on a 4-point Likert scale, and were transformed to scales from 0 to 100 according to the questionnaire guidelines [21]. For all quality of life scales, a higher score corresponds to a better quality of life or level of functioning.

### Demographic and clinical characteristics

All participants filled out a general questionnaire for demographic information which included age, smoking status (current, former, never), educational level (low, middle, high), and living situation (alone, with partner and/or children). Information on stage of disease at diagnosis (stage I, II, or III) and treatment (neo- or adjuvant chemotherapy, type of chemotherapy, hormone treatment yes/no, trastuzumab yes/no) were obtained from patients’ medical records. Chemotherapy regimens were categorized to combined and sequential regimes. Combined regimes included schemes where all different components were administered together during all cycles, such as TAC (6 × docetaxel, doxorubicin and cyclophosphamide every 3 weeks). Sequential regimes included schemes where different components were administered in different cycles such as ACP (ACP: 4 × adriamycin and cyclophosphamide every 3 weeks followed by 12 × paclitaxel weekly).

### Data analysis

Demographic, clinical variables and prevalence of taste and smell changes are presented as mean ± SD or *n* (%). We used a linear mixed model analysis to assess differences in the AHSP subscales for taste and smell and quality of life outcomes over time and between groups. Time (T1 and T2) and group (patient or comparison group) were included in the model as fixed and participants as random factor.

Analysis of covariance was used to assess associations between demographic, clinical variables (stage of disease, adjuvant/neo-adjuvant treatment, type of chemotherapy, hormone treatment, and trastuzumab) and taste and smell on both time points separately. Analysis of covariance was also used to assess the association between taste, smell, and quality of life. Relevant covariates were included in the models based on the literature and change of regression coefficient. Variables that changed the regression coefficient ≥ 10% in the adjusted model compared to the crude model were included in the final model. Final models were adjusted for age. Possible effect modifiers were assessed by including interactions in the model. Variables assessed as effect modifiers were as follows: adjuvant/neo-adjuvant treatment, type of chemotherapy (combined vs. sequential), hormone treatment (yes vs. no), and trastuzumab (yes vs. no). Statistical analyses were performed using IBM SPSS statistics version 23. A *p* value < 0.05 was considered statistically significant.

## Results

Demographic and clinical characteristics of the patient and the control group are shown in Table 1. Breast cancer patients had a slightly lower age and higher BMI than the women without breast cancer. Furthermore, the patient group had more current smokers and fewer former smokers than the comparison group. The groups were similar in education level and living situation. Most patients had a stage II tumor and were treated with adjuvant chemotherapy.

**Table 1:** Demographic and clinical characteristics of women without breast cancer (comparison group), and women with breast cancer (patients) presented as mean (SD) or *n* (%)

	Comparison *n* = 114	Patients *n* = 135
Demographic characteristics		
Age (years)	55.4 (10.2)	52.5 (9.1)
BMI (kg/m^2^)	24.9 (3.5)	26.0 (4.0)
Smoking^a^		
Current	10 (9)	21 (15)
Former	62 (54)	56 (42)
Never	42 (37)	57 (43)
Education^a^		
Lower	9 (8)	12 (9)
Middle	32 (28)	42 (31)
Higher	73 (64)	80 (60)
Living situation^a^		
Alone	15 (13)	16 (12)
With partner and/or children	99 (87)	118 (88)
Clinical characteristics		
Adjuvant chemotherapy		83 (61)
Neo-adjuvant chemotherapy		52 (39)
Stage		
I		36 (27)
II		79 (59)
III		19 (14)
Chemotherapy		
Combined treatment		62 (46)
Split treatment		73 (54)
Hormone treatment^a^		
Yes		104 (78)
No		30 (22)
Trastuzumab		
Yes		31 (23)
No		104 (77)

^a^1 missing for patient group

**Fig. 2:**
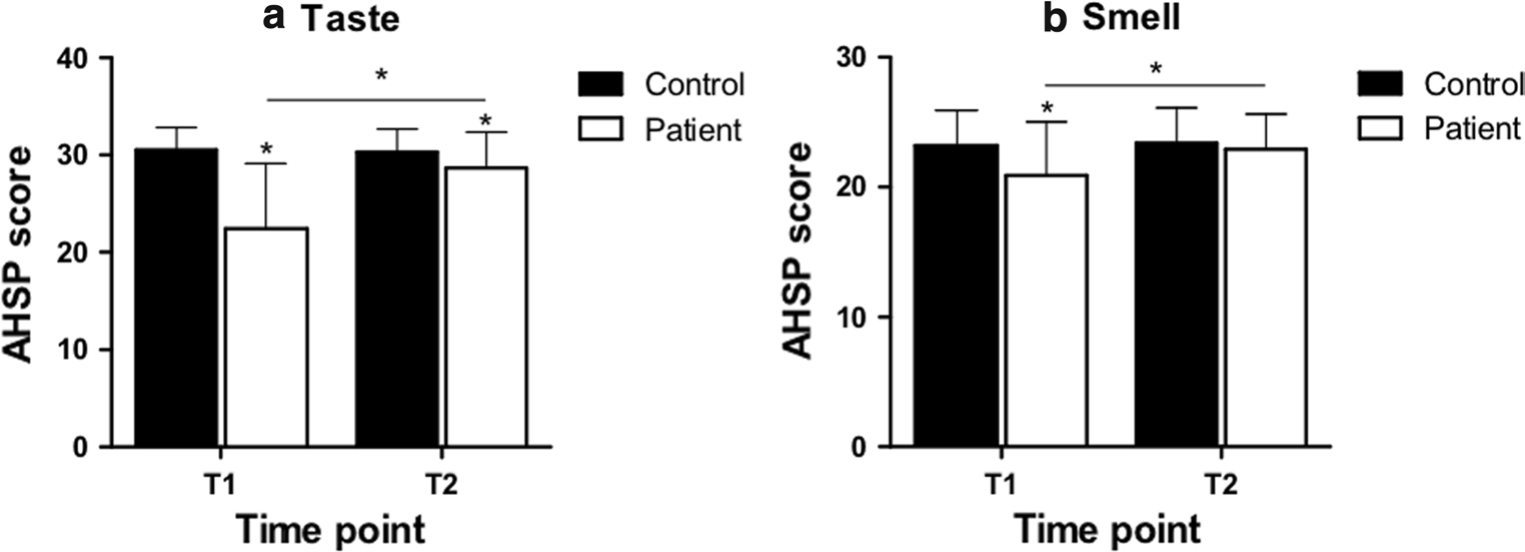
Taste and smell scores (mean ± SD) of the AHSP questionnaire over time for the patient and comparison group. T1 represents the first measurement (comparison) and shortly after chemotherapy (patients), T2 represents 6 months after the first measurement (comparison) or ~ 6 months after the end of chemotherapy (patients). *indicates a significant difference at *p* < 0.05

Both shortly after (T1) and half a year after chemotherapy (T2), breast cancer patients reported a lower taste perception compared to the comparison group (Fig. 2a). The comparison group remained stable, while, in the patient group, self-reported taste perception improved over time. Results were similar for self-reported smell perception (Fig. 2b), although there was no significant difference for smell at T2 between the patient and the comparison group.

At T1 (shortly after chemotherapy), 65% of patients reported their taste perception as worse compared to before chemotherapy, 3% reported a better taste perception and 32% reported their taste perception as unchanged. Half a year later (T2), 16% of patients reported a worse taste perception, 8% a better taste perception and 76% reported their taste to be the same as before chemotherapy.

For smell at T1, 19% of patients reported their smell perception to be worse; 16% reported a better smell perception, and 65% reported their smell to be the same as before chemotherapy. Half a year later (T2), only 3% reported a worse smell perception, 12% better, and 85% reported their smell perception to be the same as before chemotherapy.

Both shortly after (T1) and half a year after chemotherapy (T2), age, BMI, smoking status, living situation, education level, stage of disease, receiving adjuvant or neo-adjuvant treatment, type of chemotherapy and receiving hormone treatment were all not associated with self-reported taste or smell (data not shown). At T2, patients receiving trastuzumab (*n* = 31) scored 2.6 points lower on the taste scale (*β* = − 2.6, 95% CI − 4.17; − 1.08, *p* = 0.001) and 2.0 points lower smell scale (*β* = − 2.0, 95% CI − 3.12; − 0.87, *p* = 0.001) compared to patients not receiving trastuzumab (*n* = 104).

**Fig. 3:**
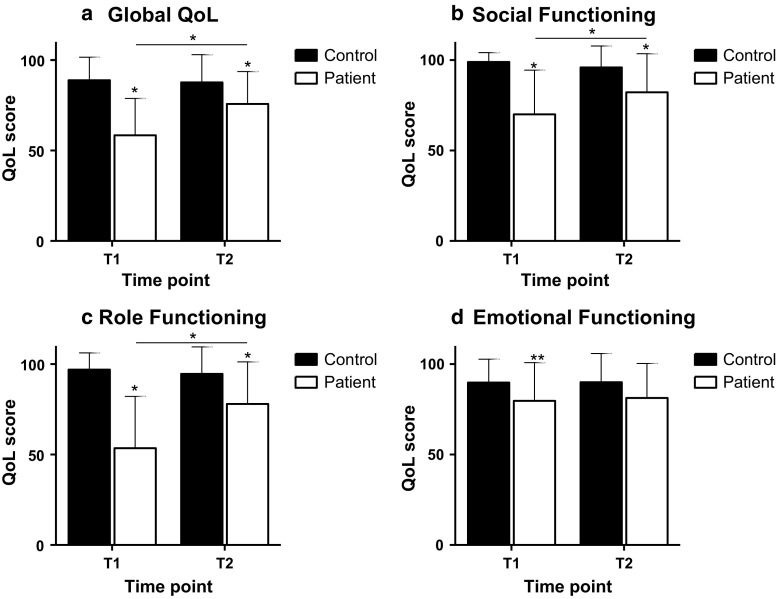
Global quality of life (**a**) and function scales (**b** social, **c** role, and **d** emotional functioning) of the EORTC-QLQ-C30 (mean ± SD) over time for the patient and comparison group. T1 represents the first measurement which for the patients was done shortly after chemotherapy; T2 is the second measurement, which took place ~ 6 months after the first measurement. *indicates a significant difference at *p* < 0.05

Global quality of life was significantly lower in the patient group compared to the comparison group at both time points (Fig. 3a), but significantly improved half a year after chemotherapy. These patterns between groups and over time were similar for role functioning and social functioning (Fig. 3b, c). For emotional functioning, patients scored lower compared to the comparison group at T1 and T2, and this did not improve half a year after chemotherapy (Fig. 3d).

### Associations between taste, smell, and quality of life shortly and half a year after chemotherapy

In patients, better reported taste and smell shortly after chemotherapy (T1) were statistically significantly associated with better global quality of life, role functioning, social functioning and emotional functioning (Table 2).

**Table 2:** The association between taste, smell, and quality of life outcomes in breast cancer patients shortly after chemotherapy (T1), adjusted for age

	β	SE	*p* value	95% CI
Taste				
Global quality of life	0.99	0.26	< 0.001	0.47; 1.51
Role functioning	1.74	0.36	< 0.001	1.03; 2.44
Social functioning	1.14	0.32	< 0.001	0.51; 1.77
Emotional functioning	0.79	0.28	< 0.005	0.24; 1.35
Smell				
Global quality of life	1.09	0.44	0.02	0.21; 1.96
Role functioning	1.50	0.62	0.02	0.27; 2.74
Social functioning	1.69	0.52	< 0.01	0.65; 2.72
Emotional functioning	1.23	0.46	< 0.01	0.32; 2.14

β represents the difference in quality of life outcome per 1 unit higher score on taste or smell as assessed with the AHSP questionnaire

Half a year after the end of chemotherapy, the association between taste, smell, and global quality of life, role functioning and social functioning, was different for patients receiving trastuzumab versus patients not receiving trastuzumab (Table 3). Only in patients who received trastuzumab, better reported taste and smell perception were statistically significantly associated with a better global quality of life; in addition, better reported taste and smell were associated with better role functioning and social functioning. In patients not treated with trastuzumab, reported taste and smell perception were not statistically significantly associated with global quality of life, role functioning, and social functioning.

**Table 3:** The association between taste, smell, and quality of life outcomes in breast cancer patients half a year after the end of chemotherapy, (T2) stratified for trastuzumab, adjusted for age

	Patients receiving trastuzumab (*n* = 31)				Patients not receiving trastuzumab (*n* = 104)			
	β	SE	*p* value	95% CI	β	SE	*p* value	95% CI
Taste								
Global quality of life	2.52	0.77	< 0.01	0.92; 4.12	0.76	0.53	0.15	− 0.29; 1.81
Role functioning	3.01	0.88	< 0.01	1.19; 4.82	0.24	0.77	0.76	− 1.29; 1.76
Social functioning	3.47	0.93	< 0.001	1.56; 5.38	0.78	0.62	0.21	− 0.46; 2.01
Smell								
Global quality of life	3.11	1.16	0.013	0.72; 5.49	0.37	0.72	0.60	− 1.06; 1.81
Role functioning	3.05	1.39	0.04	0.18; 5.92	− 0.29	1.04	0.78	− 2.36; 1.77
Social functioning	4.18	1.42	< 0.01	1.25; 7.10	0.52	0.84	0.54	− 1.16; 2.20

β represents the difference in quality of life outcome per 1 unit higher score on taste or smell as assessed with the AHSP questionnaire

## Discussion

In this study, we assessed reported taste and smell changes shortly after, and 6 months after chemotherapy in breast cancer patients compared to a group of women without breast cancer. Furthermore, we determined the association between taste and smell perception and quality of life shortly after and 6 months after chemotherapy.

In line with previous studies in breast cancer patients, we showed that taste and smell perception are altered shortly after the end of chemotherapy, but recovered in the 6 months after the end of chemotherapy for most patients [8, 9]. The prevalence of taste (68%) and smell alterations (35%) shortly after chemotherapy are also within the range described in the previous literature for taste (45–84%) and smell (5–60%) [22]. Interestingly, half a year after chemotherapy self-reported taste and smell perception were lower in patients receiving trastuzumab compared to patients not treated with trastuzumab.

We found that lower taste and smell perception were associated with lower quality of life, which is in line with previous studies in cancer patients during chemotherapy [4, 5, 23]. In addition, role and social functioning were associated with worsened taste and smell perception. Although these findings are associations, it is likely that changes in role functioning could indeed be a result of changes in taste and smell, e.g., role functioning may be affected because a partner needs to take over cooking, because cooking smells are being experienced by patients as offensive or nauseating [2, 23]. Problems with cooking are commonly reported in patients with olfactory dysfunction, as they have difficulties with smelling whether food is spoiled [24, 25]. In addition, research in the general population has shown that women with good smell function tend to have more active social lives than those with diminished smell function [26].

To the best of our knowledge, this is the first study that showed that taste or smell changes persist during the period of trastuzumab when the patients are no longer receiving chemotherapy. Our follow-up period started half a year after the end of chemotherapy, so we could not study whether these changes in taste and smell in trastuzumab-treated patients were also transient and recovered after trastuzumab ended. Therefore, a future project should assess taste and smell perception before, at several moments during and after the end of treatment with trastuzumab. This will give insight in whether these alterations might diminish or worsen over the trajectory, and whether these alterations recover after the end of treatment with trastuzumab. If future studies confirm our findings on trastuzumab and taste and smell, further mechanistic work is needed to understand how trastuzumab affects the sensory system. For chemotherapy, the general hypothesis is that it acts on rapidly dividing cells, and therapy may therefore also impact the taste and smell receptor cells that have a turnover rate of 1 week to a month [27, 28]. Objective measurements of taste and smell function could help to elucidate whether these alterations are due to actual dysfunction of the sense of taste and/or smell. Research has shown that a lower taste perception during chemotherapy in breast cancer patients is associated with a lower energy intake, specifically for protein and fat intake [17]. Potentially, patients who are treated with trastuzumab have a lower energy intake for a prolonged period of time, which might have an impact on their nutritional status.

Although we did not perform objective or baseline smell or taste measurements in the complete patient and comparison group in the present study, we did collect these data in a subgroup of patients and control women (*n* = 28 per group, as reported in [29]). Those data show that before start of chemotherapy there are no differences in self-reported smell or taste perception, or in objective smell or taste measurements, between patients and control women. This suggests that the findings reported here reflect actual changes in taste perception in the patient group, rather than differences between the groups, or alterations in the patient group already at baseline.

Six months after the end of chemotherapy, the score on taste was 2.6 points lower, and on smell 2.0 points lower in patients receiving trastuzumab than in patients not receiving trastuzumab. Even though the differences were small, there was a significant association with quality of life at this time point in the patients receiving trastuzumab, which was not present among the patients not receiving trastuzumab. This suggests that, although reported differences in taste and smell are small, they may affect quality of life.

Unfortunately, there are currently no effective interventions for taste and smell alterations in cancer patients. However, it is important to monitor these alterations over the treatment trajectory in breast cancer patients, in particular given that those changes may have an impact on quality of life and on nutritional status. After chemotherapy has ended, specifically patients who are treated with trastuzumab are a group of interest that warrants the attention of clinicians.

In conclusion, this study shows that most taste and smell alterations recover after chemotherapy for breast cancer, but importantly, not for patients who receive trastuzumab. Monitoring of changes in taste and smell may be warranted especially for patients receiving trastuzumab as they may potentially impact quality of life and nutritional status.

## Data availability

The datasets analyzed during the current study are available from the corresponding author on reasonable request.
